# Investigating the regulatory effect of Shen Qi Bu Qi powder on the gastrointestinal flora and serum metabolites in calves

**DOI:** 10.3389/fcimb.2024.1443712

**Published:** 2024-08-23

**Authors:** Haochi Yang, Jianming Ren, Peng Ji, Xiaosong Zhang, Zhanhai Mai, Chenchen Li, Nianshou Zhao, Ting Ma, Xiaopeng Zhu, Yongli Hua, Yanming Wei

**Affiliations:** ^1^ College of Veterinary Medicine, Gansu Agricultural University, Lanzhou, China; ^2^ College of Chemistry and Life Sciences, Gansu Minzu Normal University, Gannan, China; ^3^ Innovation Center for Traditional Chinese Veterinary Medicine, College of Veterinary Medicine, China Agricultural University, Beijing, China; ^4^ Zhangye Wanhe Animal Husbandry Industry Technology Development Co., Ltd, Zhangye, China

**Keywords:** calves, average daily gain, serum indicators, gastrointestinal flora, serum metabolites, correlation analysis

## Abstract

**Object:**

To investigate the effects of Shen Qi Bu Qi Powder (SQBQP) on the average daily gain, blood indexes, gastrointestinal microflora, and serum metabolites of calves.

**Methods:**

A total of 105 calves were randomly assigned to three groups (n = 35 per group): the control group (C, fed with a basal diet for 21 days) and two treatment groups (SQBQP-L and SQBQP-H, fed with the basal diet supplemented with 15 and 30 g/kg of SQBQP), respectively for 21 days. The active components of SQBQP were identified using LC-MS/MS. Serum digestive enzymes and antioxidant indices were determined by ELISA kits and biochemical kits, respectively. Serum differential metabolites were analyzed by liquid chromatography-mass spectrometry/mass spectrometry (LC-MS/MS), while flora in rumen fluid and fecal were analyzed by 16S rDNA sequencing. Further correlation analysis of gastrointestinal flora and serum metabolites of SQBQP-H and C groups were performed with Spearman’s correlation.

**Results:**

The principal active components of SQBQP mainly includes polysaccharides, flavonoids, and organic acids. Compared to the control group (C), calves in the SQBQP-H (high dose) and SQBQP-L (low dose) groups showed a significant increase in serum amylase (AMS) levels (*P*<0.001), while lipase content significantly decreased (*P*<0.05). Additionally, the average daily gain, T-AOC, and cellulase content of calves in the SQBQP-H group significantly increased (*P*<0.05). *Proteobacteria* and *Succinivibrio* in the rumen flora of the SQBQP-H group was significantly lower than that of the C group (*P*<0.05). The relative abundance of *Proteobacteria, Actinobacteria, Candidatus_Saccharibacteria, Deinococcus_Thermus, Cyanobacteria*, and *Succinivibrio* in the SQBQP-H group was significantly increased (*P*<0.05), while the relative abundance of *Tenericutes* and *Oscillibacter* was significantly decreased (*P*<0.05). Serum metabolomics analysis revealed 20 differential metabolites, mainly enriched in amino acid biosynthesis, β-alanine metabolism, tyrosine, and tryptophan biosynthesis metabolic pathways (*P*<0.05). Correlation analysis results showed that *Butyrivibrio* in rumen flora and *Oscillibacter_valericigenes* in intestinal flora were significantly positively correlated with average daily gain, serum biochemical indexes, and differential metabolite (-)-Epigallocatechin (R>0.58, *P*<0.05).

**Conclusion:**

SQBQP can promote calves weight gain and enhance health by modulating gastrointestinal flora and metabolic processes in the body.

## 1 Introduction

In light of the rapid expansion of the beef cattle breeding industry in China, the intensification degree is on the rise. The off-site fattening mode has emerged as the dominant paradigm in this context. Factors such as transportation, driving, vaccination stimuli, and heat stress negatively impact calves, leading to reduce immunity and growth performance, and increase morbidity and mortality (Deters and Hansen, 2022). To support the “Antibiotic Reduction Action” initiative by the Ministry of Agriculture and Rural Affairs of China and to promote the healthy, green, and sustainable development of the beef cattle industry, it is crucial to conduct research on the efficacy of Chinese veterinary compounds for improving calf health and growth.

Shen Qi Bu Qi Powder (SQBQP) is a self-made prescription by us, which primarily comprises natural Chinese herbal medicines, including *Radix Astragali* and *Rhizoma Atractylodis macrocephalae* and so on. The prescription has the effects of strengthening the stomach, replenishing qi, and consolidating the surface. Previous research conducted by us found that SQBQP could improve the immune index of 3-month-old calves, and could not effect on the liver and kidney function. The active ingredients of traditional Chinese medicine (TCM) regulate the immune level of animals by affecting the antioxidant level (Huo et al., 2022), digestive enzyme activity (Liu et al., 2023d; Long et al., 2020), rumen, and intestinal microbial flora (Che et al., 2022; Chen et al., 2023), thus achieving the desired health care effect. Some studies found that TCM was rich in active ingredients such as polysaccharides, flavonoids, and saponins (Zhu and Yu, 2018), which play an important role in bacteriostasis (Shi et al., 2022), anti-inflammatory (Lin et al., 2015), promoting growth (Liu et al., 2023b), and improving immunity effects (Liu et al., 2019). The medicinal and food-related TCM like *Radix Astragali* and *Rhizoma Atractylodis macrocephalae* have demonstrated extensive applications in breeding industry, including the rearing of aquatic animals, poultry, pigs, sheep, cows, and rabbits (Salazar et al., 2019; Swelum et al., 2021; Zou et al., 2022). The serum antioxidant index is a crucial indicator for assessing oxidative stress in animals. The active ingredients present in *Radix Astragali* and *Rhizoma Atractylodis macrocephalae* include polysaccharides, organic acids, and flavonoids, which have been demonstrated to effectively enhance the activity of antioxidant enzymes such as superoxide dismutase (SOD) and glutathione peroxidase (GSH-Px) in serum. These ingredients have been shown to reduce the damage caused by oxidative free radicals to cell membranes and to maintain intestinal barrier function (Hao et al., 2020; Wang et al., 2021). Serum digestive enzyme activity is a key indicator of the animal’s digestion and absorption abilities. TCM practices have been shown to enhance the activity of digestive enzymes, including pepsin, trypsin, and amylase, thus facilitating the nutrient digestion and absorption and improving the production performance (Harikrishnan et al., 2022). The rumen microbial flora plays an important regulatory role in the nutrition and metabolism of ruminants. The active ingredients in TCM have been shown to enhance feed degradation and fermentation, thereby improving the feed utilization by regulating the structure of gastrointestinal microbial flora (Kholif and Olafadehan, 2021; Harikrishnan et al., 2022; Rabee et al., 2024). These ingredients selectively promote the growth of beneficial bacteria, inhibit harmful bacteria, maintain rumen environment stability, and enhance body immunity (Liu et al., 2023b; Ma et al., 2023). Analyzing serum metabolites provides a deeper understanding of the effects of TCM on the animal metabolism and elucidates the comprehensive multi-component, multi-channel, and multi-target mechanisms of TCM action (Liu et al., 2023a).

In this study, SQBQP was used to feed calves, and its effects on the growth performance, serum antioxidant capacity, digestive enzyme activity, rumen and intestinal microflora, and serum metabolism of calves were analyzed. The aim was to systematically investigate the mechanisms of promoting calf health and weight gain of SQBQP, thereby support its effective application in the healthy breeding of calves.

## 2 Materials and methods

### 2.1 Preparation of Shen Qi Bu Qi powder

*Radix Astragali, Radix Codonopsis pilosulae, Radix Saposhnikoviae divaricatae, and Rhizoma Atractylodis macrocephalae* were crushed. Then they were passed through a 100-mesh sieve and mixed in proportion to create the final preparation.

### 2.2 LC-MS/MS analysis of SQBQP samples

The active components of SQBQP were subjected to analysis by Waters 2D UPLC (Waters, USA) in conjunction with a Q Exactive high-resolution mass spectrometer (Thermo Fisher Scientific, USA). Chromatographic conditions were as follows: the chromatographic column used was a Hypersil GOLD a Q column (100*2.1 mm, 1.9 μm, Thermo Fisher Scientific, USA). The mobile phases employed were 0.1% formic acid in water (liquid A) and 100% acetonitrile containing 0.1% formic acid (liquid B). The elution gradients were as follows: The mobile phases were as follows: 0-2 min, 5% B; 2-22 min, 5%-95% B; 22-27 min, 95% B; 27.1-30 min, 5% B. The flow rate was 0.3 mL/kg. The column temperature was maintained at 40°C, the flow rate was set at 0.3 mL/min, and the injection volume was fixed at 5 μL. Mass spectrometry: The primary and secondary mass spectrometry data were collected using a Q Exactive mass spectrometer (Thermo Fisher Scientific, USA). The mass-to-charge ratio range for mass spectrometry scanning was 150-1500, with a primary resolution of 70,000, an AGC of 1e^6^, and a maximum injection time (IT) of 100 ms. The top three parent ions were selected for fragmentation, and secondary information was subsequently collected. The secondary resolution was 35,000, the AGC was 2e^5^, the maximum injection time (IT) was 50 ms, and the stepped voltage was set to 20, 40, and 60 eV. The settings for the ion source (ESI) were as follows: the sheath gas flow rate was 40, the auxiliary gas flow rate was 10, the spray voltage (kV) was 3.80 for positive ion mode, and 3.20 for negative ion mode, the capillary temperature was 320°C, and the temperature of the auxiliary gas heater was 350°C. The TCMSP database was used to compare the results of LC-MS/MS detection.

### 2.3 Experimental animals

A total of 105 three-month-old Simmental hybrid bull calves with a mean body weight of 122.55 ± 15.01 kg were randomly assigned to three groups, with 35 calves in each group. There was no significant difference in the body weight of the calves among the three groups. This experiment was conducted at the beef cattle farm of Wanhe Grass Livestock Industry Science and Technology Development Co., Ltd. in Zhangye City, Gansu Province, China. Calves in the control group (C) were fed with a basal diet daily (**Table 1**) for 21 days. Calves in the SQBQP-L group were fed with a basal diet supplemented with 15 g/kg of SQBQP for 21 days, and calves in the SQBQP-H group were fed with a basal diet supplemented with 30 g/kg of SQBQP for 21 days. The calves were fed twice daily, at 7:00-8:00 am and 2:30-3:30 pm, taking food and water freely.

**Table 1:** Composition of basic diet (%).

Item	Contents (%)
Corn concentrate	
ADF	4.11
NDF	9.44
P	0.56
DM	88.95
CP	11.56
EF	3.21
Ash	5.8
Ca	0.46
Na	0.644
Mg	0.26
Wheat straw	
ADF	50.75
NDF	72.58
P	0.16
DM	91.15
CP	2.42
EF	1.91
Ash	9.15
Ca	0.2

ADF, acid detergent fiber; NDF, neutral detergent fiber; DM, dry matter; CP, crude protein; EE, crude fat; Ash, crude ash.

### 2.4 Sample collection

At the end of the experiment, the blood (10 mL) was collected from the tail vein of all experimental animals before morning feeding. The serum was separated by centrifugation at 3500 rpm for 15 mins and stored at -80°C. Six calves were randomly selected from each group for ruminal fluid collection, which was filtered and stored in a refrigerator at -80°C. Additionally, 14 calves were randomly selected from each group, and approximately 5 g of fresh feces were collected and stored at -80°C.

### 2.5 Average daily gain and daily feed intake of calves

Initial and final weights were recorded before and after the test and the daily weight gain was calculated.


Average daily gain of calves=(body weight of each calf after the test−body weight of each calf before the test)/feeding days.

Feed intake=total daily feed intake per group/number of calves per group.

### 2.6 Detection of serum antioxidant and digestive enzyme indexes

Four Enzyme-linked immunosorbent assay kits (Shanghai Enzyme-linked Biological Co., Ltd.) were used to determine amylase (AMS, YJ921025), cellulase (Cellulase, YJ551029), protease (Protease, YJ361025), and lipase (Lipase, YJ295405) in calf serum. In addition, serum total antioxidant capacity (T-AOC, A015-2-1), total superoxide dismutase (T-SOD, A001-1-2), malondialdehyde (MDA, A003-1), catalase (CAT, A007-1-1), and reduced glutathione (GSH, A006-2-1) activities were measured using the biochemical kits (Nanjing Jiancheng Bioengineering Institute, Nanjing, China).

### 2.7 Detection of rumen fluid and fecal samples

A total of six rumen fluid samples and fourteen fecal samples were selected from each group for the gastrointestinal flora detection. For PCR amplification, 30 ng of qualified genomic DNA and corresponding fusion primers were used to configure the PCR reaction system and set the PCR reaction parameters. The PCR amplification products were purified using Agencourt AMPure XP magnetic beads, dissolved in the elution buffer, labeled, and the library construction was completed. The Agilent 2100 Bioanalyzer was used to determine the fragment range and concentration of the library. The qualified library was sequenced on a sequencer based on the size of the inserted fragment. After data filtering, the remaining high-quality clean data is used for post-analysis. Through the overlapping relationship between reads, reads are spliced into tags. Tags were clustered into OTUs and compared with the database and species annotation. Based on the OTU and annotation results, sample species complexity analysis and intergroup species difference analysis were performed.

### 2.8 Serum metabolomics analysis

A total of six serum samples were selected from each group for detecting serum metabolites. Metabolites were separated and detected by Waters 2D UPLC (Waters, USA) and Q Exactive high-resolution mass spectrometer (Thermo Fisher Scientific, USA). The chromatographic column used was a BEH C18 chromatographic column (1.7 μm 2.1 * 100 mm, Waters, USA). In the positive ion mode, the mobile phase consisted of 0.1% aqueous formic acid solution (A) and 100% methanol (B) containing 0.1% formic acid. In the negative ion mode, the mobile phase consisted of 10 mM ammonium formate aqueous solution (A) and 95% methanol (B) containing 10 mM ammonium formate. The following gradient was used for elution: 0-1 min, 2% B solution; 1~9 min, 2%~98% B solution; 9~12 min, 98% B solution; 12~12. 1 min, 98% B solution~2% B solution 12. 1~15 min, 2% B liquid. The flow rate was 0.35 mL/min, the column temperature was 45°C and the injection volume was 5 μL. Q Exactive mass spectrometer (Thermo Fisher Scientific, USA) was used to collect primary and secondary mass spectrometry data. The MS scan mass-to-nucleus ratio range was 70-1050, the first order resolution was 70,000, the AGC was 3e^6^ and the maximum injection time (IT) was 100 ms. According to the parent ion strength, Top3 was selected for fragmentation, and secondary information was collected. The secondary resolution was 17,500, the AGC was 1e^5^, the maximum injection time (IT) was 50 ms and the fragmentation energy (stepped once) was set to 20, 40, 60 eV. The ion source (ESI) parameters were set as follows: Sheath gas flow rate was 40, auxiliary gas flow rate was 10, spray voltage (| KV |) was 3.80 in positive ion mode, 3.20 in negative ion mode, capillary temperature was 320°C, auxiliary gas heater temperature was 350°C.

### 2.9 Statistical methods

Statistical analysis was performed using the GraphPad. Prism. 9.5 software. The data were presented in the form of X ± SEM, and T-test and one-way analysis of variance were used for group comparison. *P*<0.05 indicated a significant difference, and *P*<0.01 indicated an extremely significant difference.

## 3 Results

### 3.1 LC-MS/MS analysis of SQBQP sample analysis

**Figure 1:**
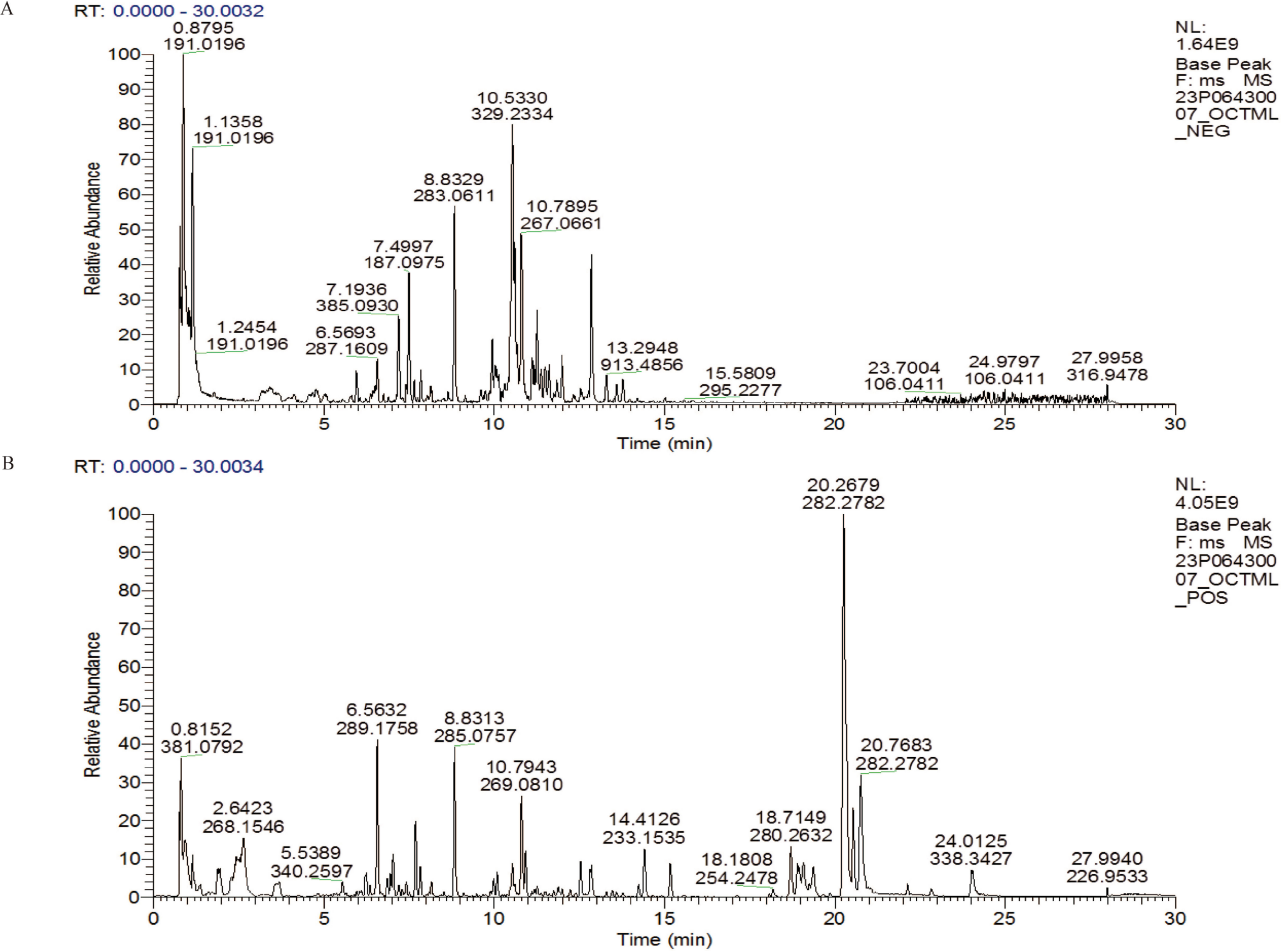
LC-MS/MS total ion chromatogram in SQBQP. **(A)** Negative ion mode diagram; **(B)** Positive ion mode diagram.

The TCMSP database was used to compare the results of LC-MS/MS detection. The results showed that 27 active components were identified in SQBQP, including polysaccharides, amino acids, flavonoids, nucleic acids, organic acids, and alcohols. The top three components were polysaccharides, flavonoids, and organic acids (**Figure 1**, **Table 2**).

**Table 2:** A detailed characterization of the components included in the SQBQP.

NO.	Name	Source of compound Chinese medicine	RT/min	Molecular Weight (da)	Delta Mass (ppm)
1	Adenine	*Radix Astragali, Radix Codonopsis pilosulae, Radix Saposhnikoviae divaricatae, Rhizoma Atractylodis macrocephalae*	0.939	135.05444	-0.401931342
2	Citric acid	*Radix Astragali, Rhizoma Atractylodis macrocephalae*	1.16	192.0268	-1.053490593
3	Galactitol	*Radix Astragali, Rhizoma Atractylodis macrocephalae*	1.047	182.07912	0.442739992
4	L-isoleucine	*Radix Astragali*	1.344	131.09465	0.129853029
5	Maleic acid	*Radix Astragali*	0.922	116.01091	-0.432249479
6	Lactose	*Radix Astragali*	1.315	388.1216	134472.9735
7	Succinic acid	*Radix Astragali*	1.24	118.02659	-0.185995698
8	Sucrose	*Radix Astragali*	0.966	342.11611	-0.291907329
9	Thymidine	*Radix Astragali*	1.786	242.09003	-0.991738137
10	Uridine	*Radix Astragali*	1.166	244.06908	-1.867001845
11	D-erythronic acid	*Radix Astragali*	1.242	136.03712	-0.355699107
12	Mucic acid	*Radix Astragali*	0.788	210.03741	-0.725651461
13	Mannitol	*Radix Astragali*	0.956	182.07899	-0.278588206
14	Ferulic acid	*Radix Saposhnikoviae divaricatae*	5.527	194.05797	0.308934059
15	D-(+)-trehalose	*Radix Saposhnikoviae divaricatae*	1.35	342.1162	-0.045956437
16	L-sorbose	*Radix Saposhnikoviae divaricatae*	0.832	180.06329	-0.54807851
17	D-(+)-glucosamine	*Radix Saposhnikoviae divaricatae*	0.935	179.07925	-0.660385374
18	Naringenin	*Radix Codonopsis pilosulae, Radix Saposhnikoviae divaricatae*	9.55	272.06819	-1.024026333
19	Daidzein	*Radix Codonopsis pilosulae, Rhizoma Atractylodis macrocephalae*	8.393	254.05778	-0.503055877
20	D-glutamic acid	*Radix Codonopsis pilosulae*	0.998	147.0531	-0.377546123
21	D-proline	*Radix Codonopsis pilosulae*	0.977	115.06327	-0.522967837
22	Eriodictyol	*Radix Codonopsis pilosulae*	8.123	288.06448	3.794530125
23	Eupatilin	*Radix Codonopsis pilosulae*	11.547	344.08954	-0.190103823
24	1 3-dicaffeoylquinic acid	*Rhizoma Atractylodis macrocephalae*	5.631	516.12629	-0.933028349
25	Isochlorogenic acid b	*Rhizoma Atractylodis macrocephalae*	6.958	516.12627	-0.983478089
26	3 5-dicaffeoylquinic acid	*Rhizoma Atractylodis macrocephalae*	7.136	516.12619	-1.138324133

### 3.2 Effects of the SQBQP on the average daily gain and the daily feed intake of calves

**Figure 2:**
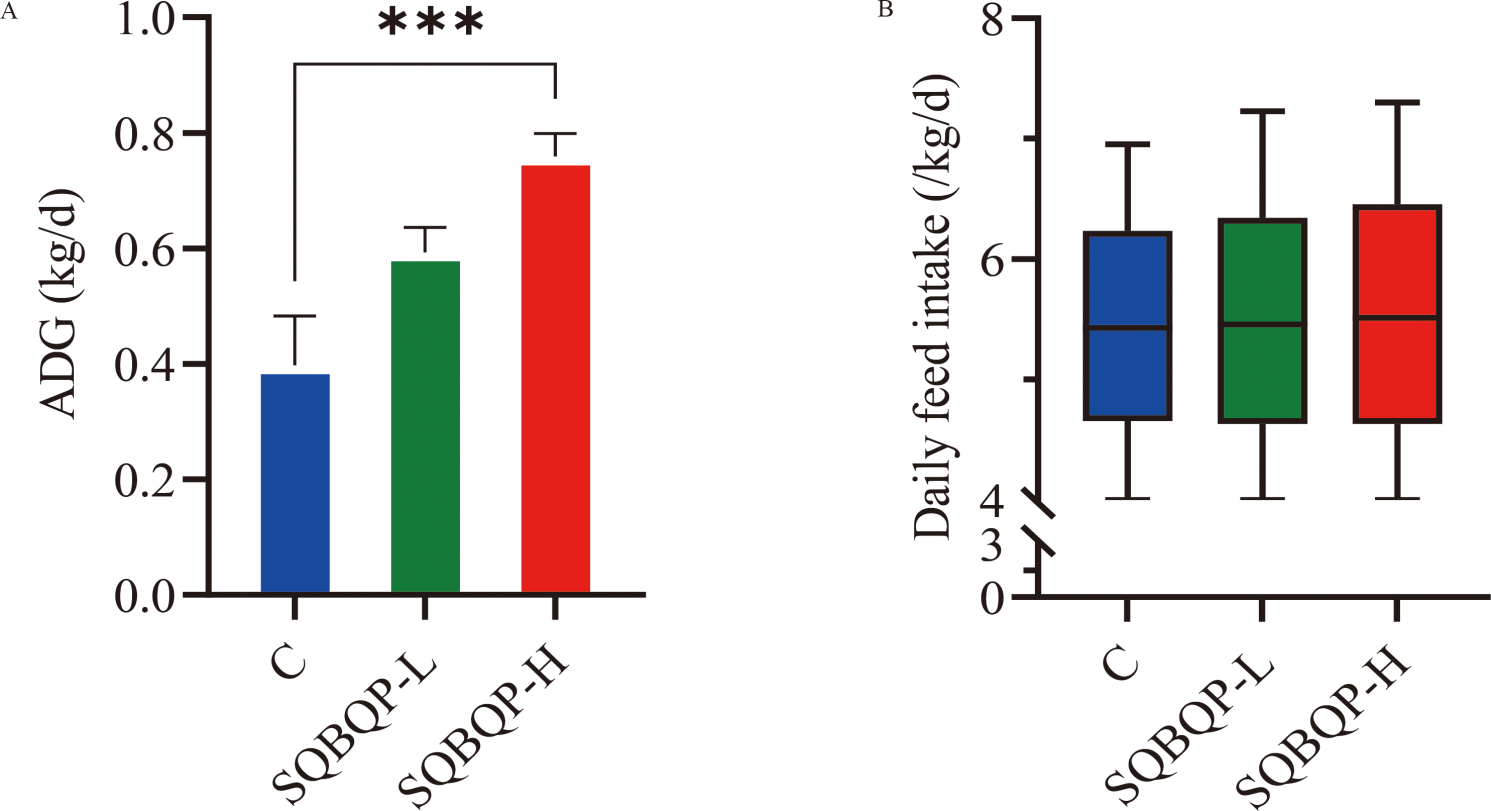
Effects of SQBQP on the average daily gain and daily feed intake of calves. **(A)** Average daily gain, **(B)** Daily feed intake. The date are expressed as SEM ± mean (n=35) and asterisks indicate significant differences (^∗∗∗^ *P*<0.001).

The average daily gain (ADG) of calves in the SQBQP-H group was significantly higher than that in the C group (**Figure 2A**). There was no significant change in the daily feed intake of calves between groups (**Figure 2B**). It was concluded that SQBQP could significantly increase the average daily gain of calves (*P*<0.05).

### 3.3 Effects of the SQBQP on the antioxidant indexes of the serum of calves

**Figure 3:**
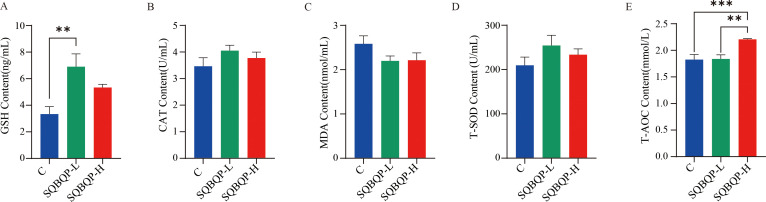
Effect of SQBQP on the serum antioxidants in calves. **(A)** GSH content, **(B)** CAT content, **(C)** MDA content, **(D)** T-SOD content, **(E)** T-AOC content. The data were expressed as SEM ± mean (n=6), and the asterisk indicated that there were significant differences according to one-way analysis of variance (^∗∗^ *P*<0.01, ^∗∗∗^ *P*<0.001).

By measuring the antioxidant indices such as GSH, CAT, and T-SOD in the calf serum, it was found that compared with the control group (C), the GSH content in the SQBQP-L group was significantly increased (*P*<0.01), and the T-AOC content in the SQBQP-H group was significantly increased (*P*<0.001). Both CAT and T-SOD showed an increasing trend, but the difference was not significant (*P*>0.05). MDA showed a decreasing trend, but the difference was not significant (*P*>0.05). These results indicated that SQBQP could improve the antioxidant capacity of calves (**Figures 3A–E**).

### 3.4 Effects of the SQBQP on serum digestive enzymes of calves

**Figure 4:**
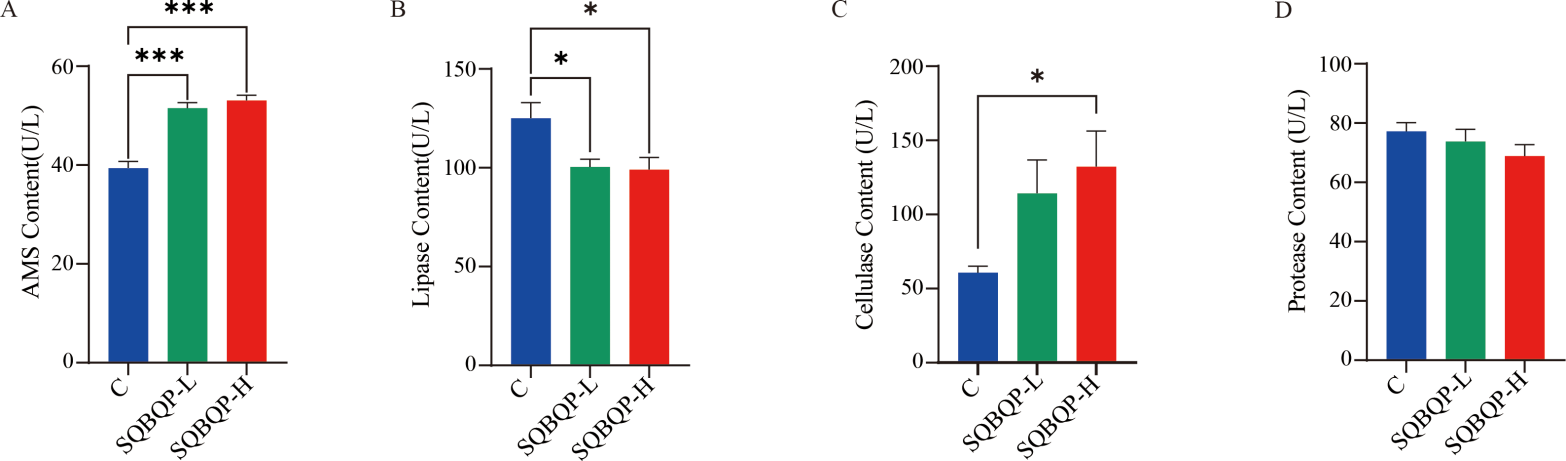
Effects of SQBQP on serum digestive enzymes in calves. **(A)** AMS content, **(B)** Lipase content, **(C)** Cellulase content, **(D)** Protease content. The data were expressed as the standard error of the mean SEM ± mean (n=6), with the asterisk indicating that there were significant differences according to one-way analysis of variance (^∗^ *P*<0.05, ^∗∗∗^ *P*<0.001).

The contents of AMS, lipase, cellulase, and protease digestive enzymes in the calf serum were measured. Compared to the control group (C), the contents of cellulase (*P*<0.05) and AMS (*P*<0.001) were significantly increased in the SQBQP-H group. The content of cellulase in SQBQP-L group had an increasing trend, but the difference was not significant (*P*>0.05). A significant decrease in the lipase content (*P*<0.05) was observed in both the SQBQP-L and SQBQP-H groups. The results demonstrated that SQBQP significantly enhanced the digestive capacity of calves (**Figures 4A–D**).

### 3.5 Effects of SQBQP-H on the rumen and intestinal flora of calves

#### 3.5.1 Effects of SQBQP-H on α and β diversity, phylum and genus level richness of rumen microbiota in calves

**Figure 5:**
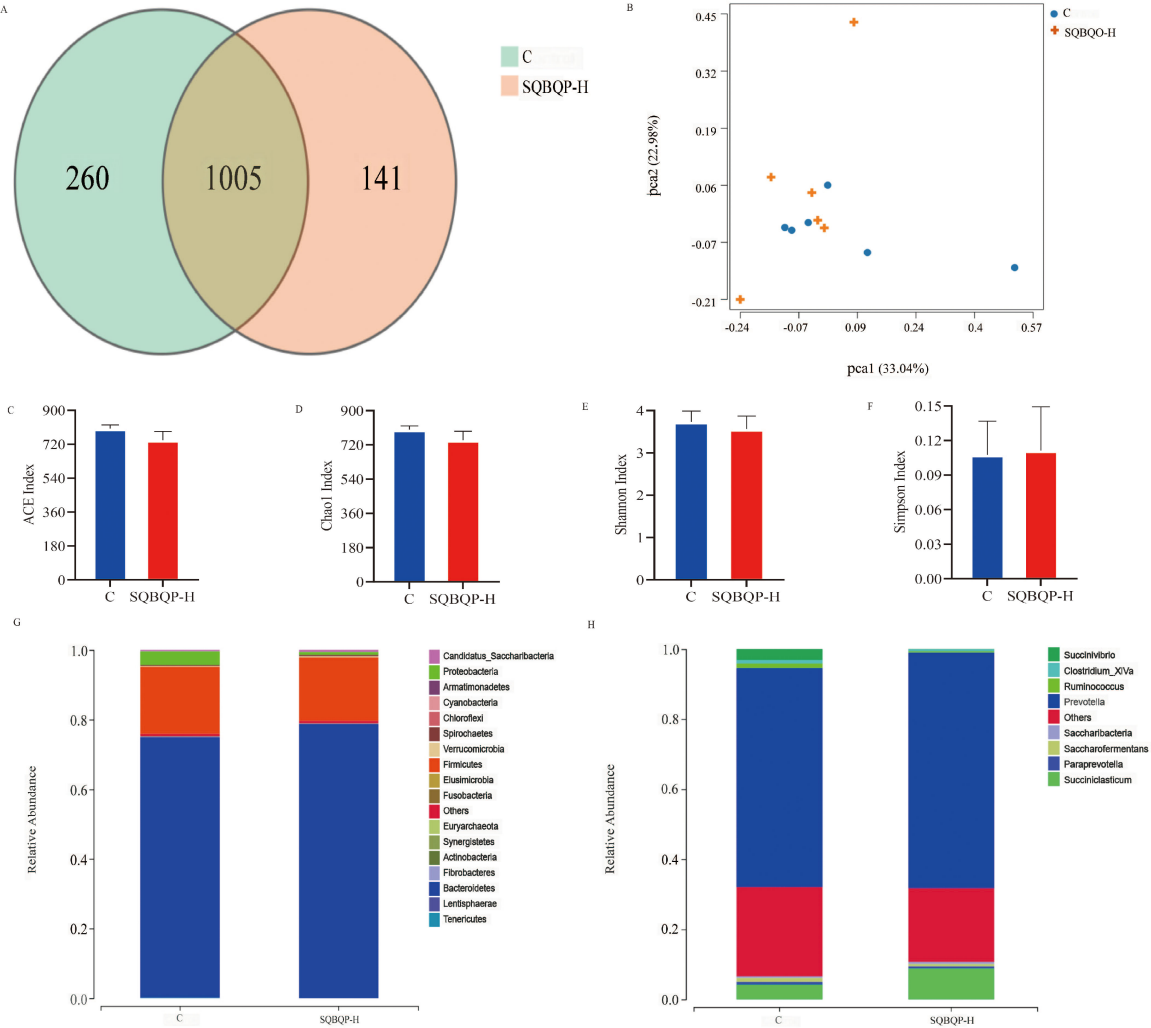
Effects of SQBQP-H on the rumen microflora composition in calves. **(A)** Venn diagram, **(B)** PCoA diagram, **(C)** Chao 1 index, **(D)** ACE index, **(E)** Shannon index, **(F)** Simpson index, **(G)** Phylum level, **(H)** Genus level (n=6).

The total number of operational taxonomic units (OUT) in group C was 1265, while the total number of OUTs in group SQBQP-H was 1146. Principal coordinate analysis (PCoA) demonstrated a certain degree of similarity between the SQBQP-H group and the C group (**Figures 5A, B**). The analysis of α and β diversity accurately reflected the species and structural diversity of the rumen microbial community. Compared to the C group, there were no significant difference in the rumen microbial Chao1, ACE, Shannon, and Simpson indexes of calves in the SQBQP-H group (**Figures 5C–F**, *P*>0.05). Furthermore, no significant differences were observed between the two groups in the core microbiota at the phylum and genus levels (**Figures 5G, H**, *P*>0.05). However, it was observed that the SQBQP-H group exhibited a significant reduction in the relative abundance of *Succinivibrio* and *Butyrivibrio* at the genus level (**Figure 5H**, *P*<0.05). The detection of rumen microbial flora revealed that SQBQP down-regulated the relative abundance of certain microbial flora without affecting the core microbial flora in the rumen of calves. It resulted in maintaining a dynamic balance of rumen microbial flora and promoting forage digestion and absorption in the rumen.

#### 3.5.2 Effects of SQBQP-H on α and β diversity, phylum and genus richness of intestinal flora in calves

**Figure 6:**
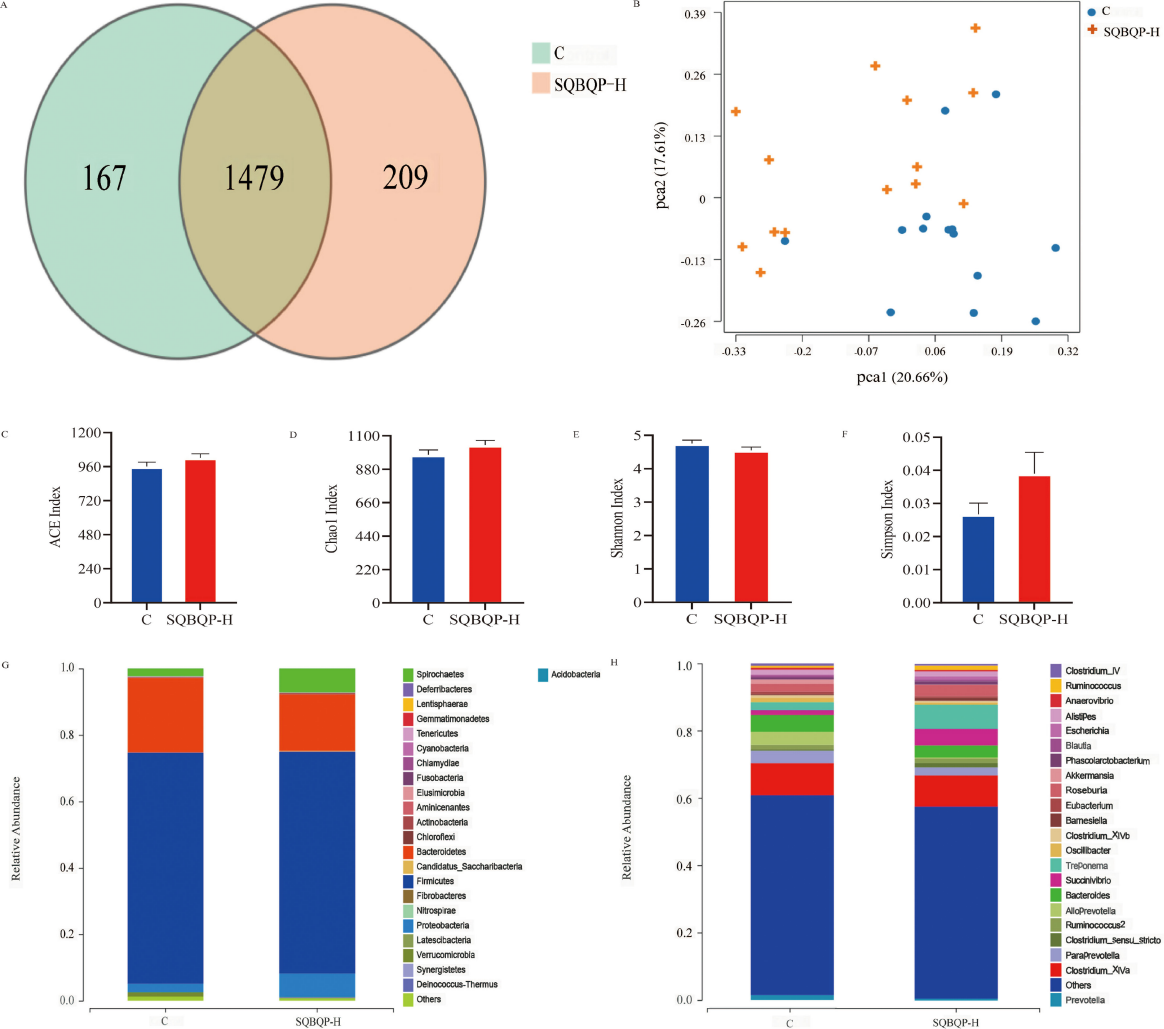
Effects of SQBQP-H on the composition of intestinal flora in calves. **(A)** Venn diagram, **(B)** PCoA diagram, **(C)** Chao 1 index, **(D)** ACE index, **(E)** Shannon index, **(F)** Simpson index, **(G)** Phylum level, **(H)** Genus level (n=14).

The total number of operational taxonomic units (OUT) in group C was 1646, while the total number of OUTs in group SQBQP-H was 1688. Principal coordinate analysis (PCoA) demonstrated a certain degree of similarity between the SQBQP-H group and the C group (**Figures 6A, B**). The α and β diversity analysis of the two groups accurately reflected the species and structural diversity of the intestinal microbial community. Compared to the C group, there were no significant differences in the Chao1, ACE, Shannon, and Simpson indexes of rumen microorganisms in the SQBQP-H group (**Figures 6C–F**, *P*>0.05). However, the relative abundance of *Proteobacteria*, *Actinobacteria*, *Candidatus_Saccharibacteria*, *Deinococcus_Thermus*, and *Cyanobacteria* at the phylum level in TOP10 of the SQBQP-H group was significantly increased compared to the C group (**Figure 6G**, *P*<0.05). Conversely, the relative abundance of *Tenericutes* was significantly decreased (**Figure 6G**, *P*<0.05). At the genus level, the relative abundance of *Succinivibrio* in the TOP10 of the SQBQP-H group was significantly increased, while that of *Oscillibacter* was significantly decreased (**Figure 6H**, *P*<0.05). The results demonstrated that SQBQP-H not only maintained the steady state of rumen microbial flora in calves but also reduced the relative abundance of harmful intestinal flora. It increased the growth and reproduction of beneficial flora, promoted the digestion and absorption of nutrients, and improved the energy utilization and average daily gain.

### 3.6 Effects of SQBQP-H on serum metabolites of calves

**Figure 7:**
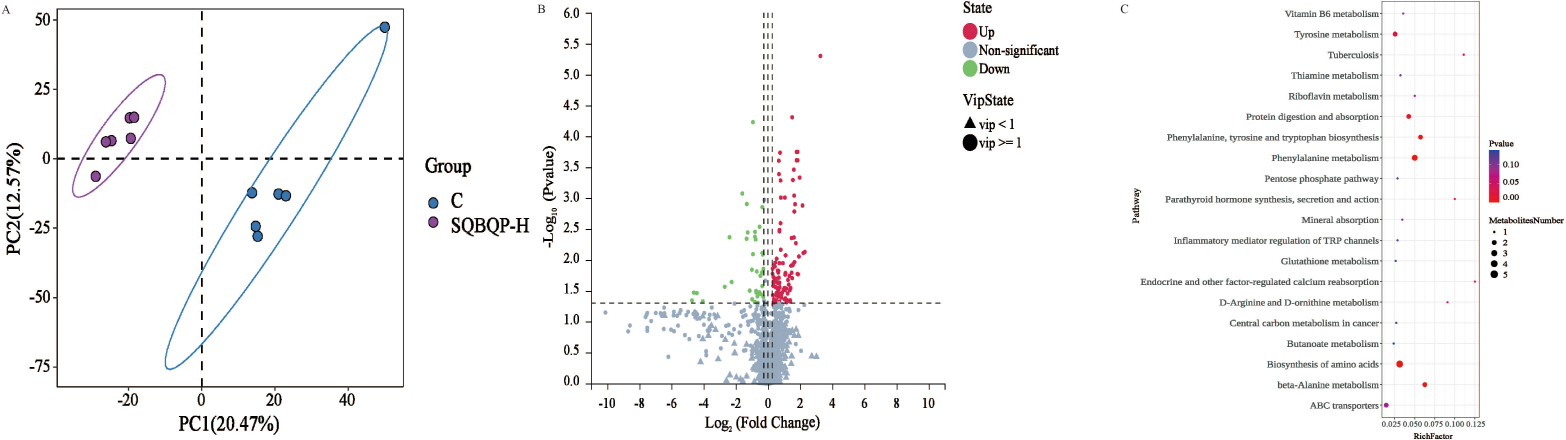
Effects of SQBQP-H on the serum metabolites in calves. **(A)** PLS-DA score plot of orthogonal partial least squares discriminant analysis of serum metabolites in the C and SQBQP-H groups, **(B)** Volcano map, each point represents a metabolite. The significantly upregulated metabolites were indicated by red dots, while the downregulated metabolites were indicated by green dots, and the metabolites with no significant difference were indicated by grey, **(C)** Kyoto Encyclopedia of Genes and Genomes (KEGG)-enriched serum differential metabolites. The color of the spot indicates the P value. The greater the degree of red, the more significant the enrichment. The size of the spots represents the number of different metabolites that are enriched (n=6).

Partial least squares discriminant analysis (PLS-DA) revealed a clear distinction between the two groups (**Figure 7A**), indicating that SQBQP-H significantly altered the serum metabolite profile. The heat map demonstrated the differential metabolites aggregated between the control group (C) and the SQBQP-H group, revealing significant metabolic differences. Furthermore, the overall distribution of differential metabolites is illustrated in the volcano plot (**Figure 7B**). In both positive and negative ion modes, a total of 150 metabolites exhibited significant changes. Among these, 108 metabolites were up-regulated, such as (-)-Epigallocatechin, Trans-cinnamaldehyde, and Trans-picerol. Additionally, 42 metabolites were down-regulated, such as arginine and Glycine-DL-phenylalanine.

The impact of SQBQP on the metabolic pathway of calves was investigated using KEGG pathway analysis. The top20 metabolic pathways of calves in the SQBQP-H group were found to be affected, as illustrated in **Figure 7C**. The analysis results revealed that SQBQP could regulate the serum metabolic pathways in calves including amino acid biosynthesis, β-alanine metabolism, phenylalanine, tyrosine and tryptophan biosynthesis, protein digestion and absorption, ABC transporters, and other metabolic pathways.

### 3.7 Correlation analysis between gastrointestinal flora and average daily gain and serum indexes

**Figure 8:**
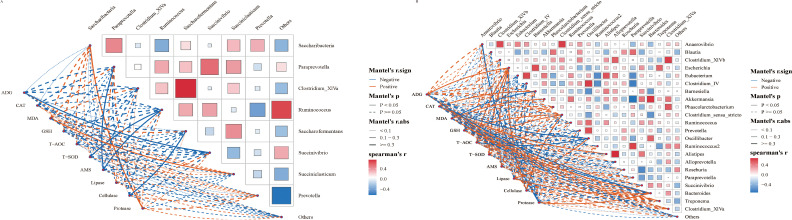
Correlation analysis between gastrointestinal flora and average daily gain and serum indexes. **(A)** Correlation between rumen flora and average daily gain and serum indicators, **(B)** Correlation between intestinal flora and average daily gain and serum indicators. The red and blue lines represent positive correlation and negative correlation, respectively. The thicker the line is, the stronger the correlation is. *P*<0.05 is the solid line.

The Spearman correlation method was employed to further investigate the relationship between gastrointestinal flora and average daily gain (ADG) and serum indexes. Overall, the correlation between gastrointestinal microbiota and ADG and serum indexes exhibited changes following the SQBQP intervention in calves (**Figures 8A, B**). The SQBQP intervention influenced weight gain and serum antioxidant and digestive enzyme indexes by regulating the homeostasis of gastrointestinal microbiota in calves. The study found that the reduction in *Succinivibrio* in the rumen microbial community of calves following the SQBQP intervention was negatively correlated with ADG (R<-0.6, *P*<0.05) (**Figure 8A**). Additionally, in SQBQP-treated calves, *Oscillibacter* was negatively correlated with CAT, T-AOC (R<-0.6, *P*<0.05), ADG, GSH, and Cellulase (R<-0.5, *P*> 0.05). Furthermore, *Oscillibacter* was found to be positively correlated with lipase (R>0.6, *P*<0.05) (**Figure 8B**). The results demonstrated that SQBQP could significantly enhance the average daily gain and serum indices of calves by regulating the relative abundance of *Succinivibrio* in rumen bacteria and *Oscillibacter* in intestinal bacteria. This further indicated that SQBQP could be employed as a traditional Chinese medicine compound to promote the growth and development of calves.

### 3.8 Correlation analysis between rumen flora and intestinal flora

**Figure 9:**
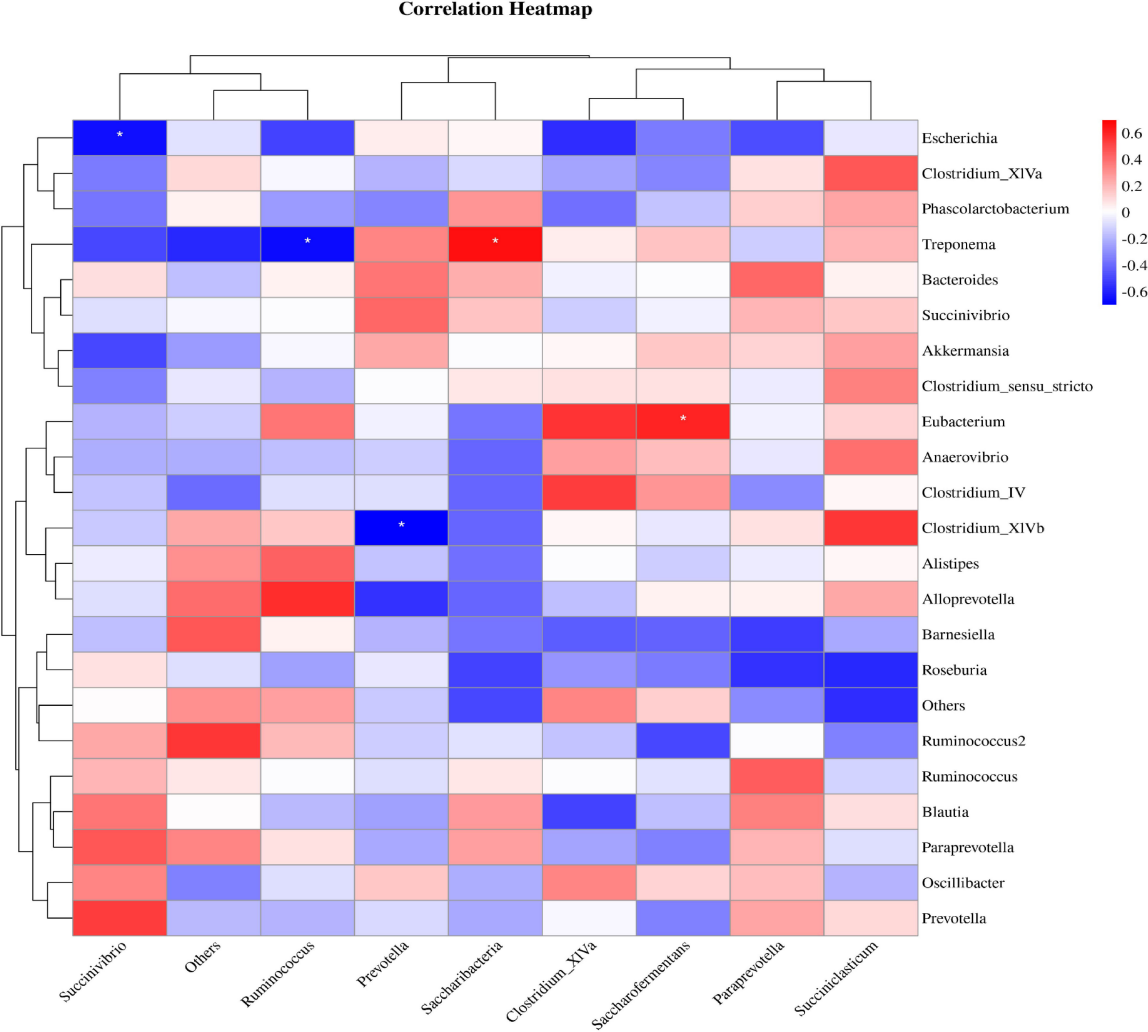
Correlation analysis between rumen flora and intestinal flora. Red indicates a positive correlation, blue indicates a negative correlation, and * represents *P*<0.05.

The correlation analysis results between the level of rumen flora and intestinal flora in calves were illustrated in **Figure 9**. It was demonstrated that *Succinivibrio* in the intestinal flora exhibited a positive correlation with *Prevotella* in the rumen flora (R>0.4, *P*>0.05). This data indicated a correlation between different bacteria in the gastrointestinal tract of calves, suggesting that one bacteria can indirectly influence the growth of another bacteria in the different internal environments.

### 3.9 Correlation analysis between serum metabolites and average daily gain and serum indexes

**Figure 10:**
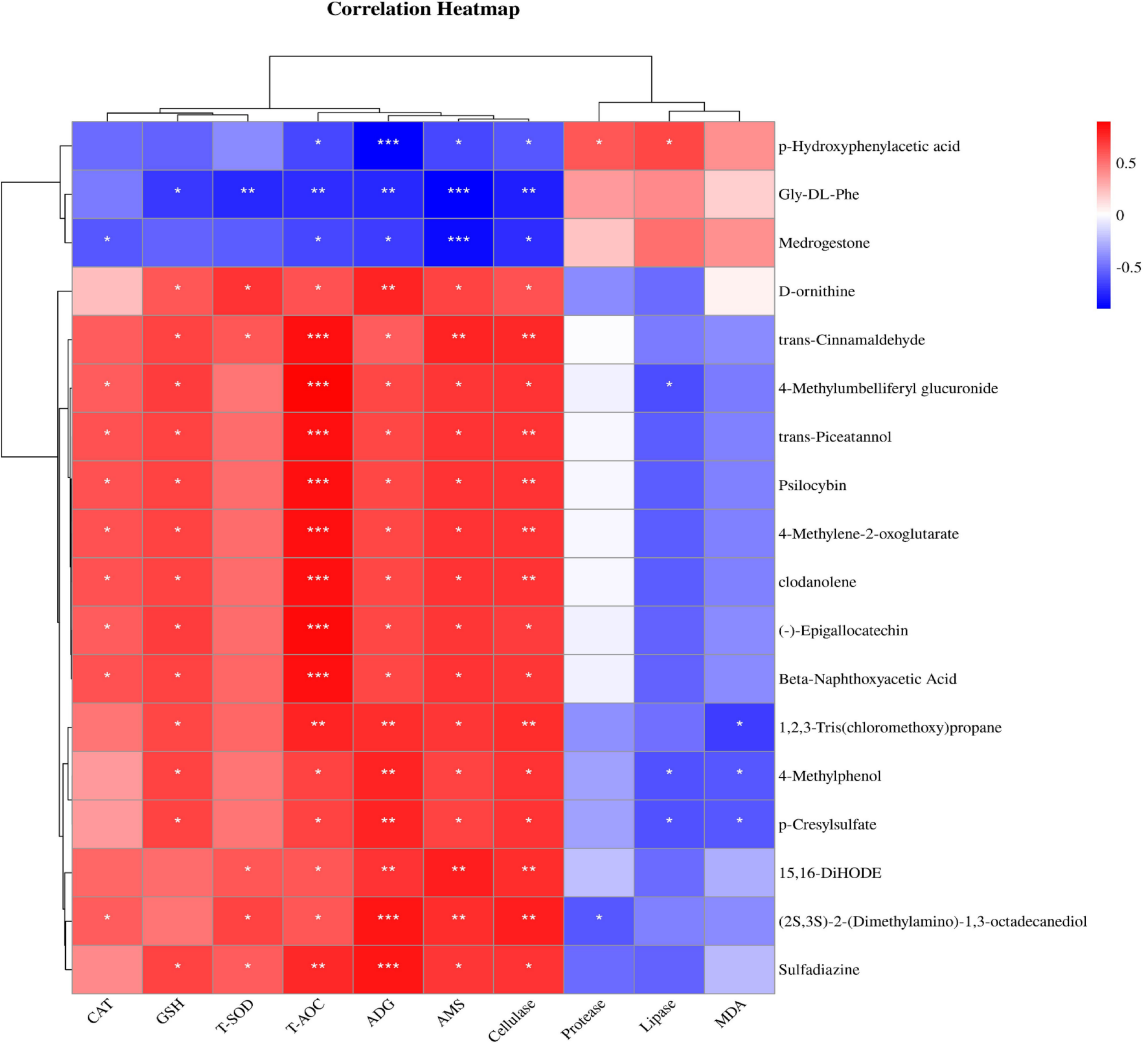
Correlation analysis between serum metabolites and average daily gain and serum indexes. Red represents positive correlation, green represents negative correlation, * represents *P*<0.05, ** represents *P*<0.01, *** represents *P*<0.001.

The results of correlation analysis between serum metabolites, average daily gain (ADG), and serum indices showed that metabolites were correlated with weight gain and serum indices after the SQBQP intervention (**Figure 10**). This study found that the serum metabolite (-)-Epigallocatechin was positively correlated with T-AOC, AMS, cellulase, GSH, ADG, and CAT (R>0.58, *P*<0.05). Trans-cinnamaldehyde was found to be positively correlated with T-AOC, AMS, cellulase, GSH, T-SOD and ADG (R>0.58, *P*<0.05). Trans-piceatannol was positively correlated with T-AOC, cellulase, AMS, GSH, ADG, and CAT (R>0.6, *P*<0.05). P-hydroxyphenylacetic acid demonstrated a positive correlation with lipase, protease, and malondialdehyde (R>0.4, *P*<0.05) while exhibiting a negative correlation with ADG, AMS, T-AOC, and cellulase (R<-0.55, *P*<0.05). Gly-DL-phenylalanine was found to be negatively correlated with AMS, cellulase, T-SOD, ADG, T-AOC, and GSH (R<-0.6, *P*<0.05). The results indicated that SQBQP could significantly enhance ADG and serum indices of calves by regulating the content of various metabolites. Consequently, SQBQP can be employed as a traditional Chinese medicine compound to enhance the growth and development of calves.

### 3.10 Correlation analysis between serum metabolites and gastrointestinal flora

**Figure 11:**
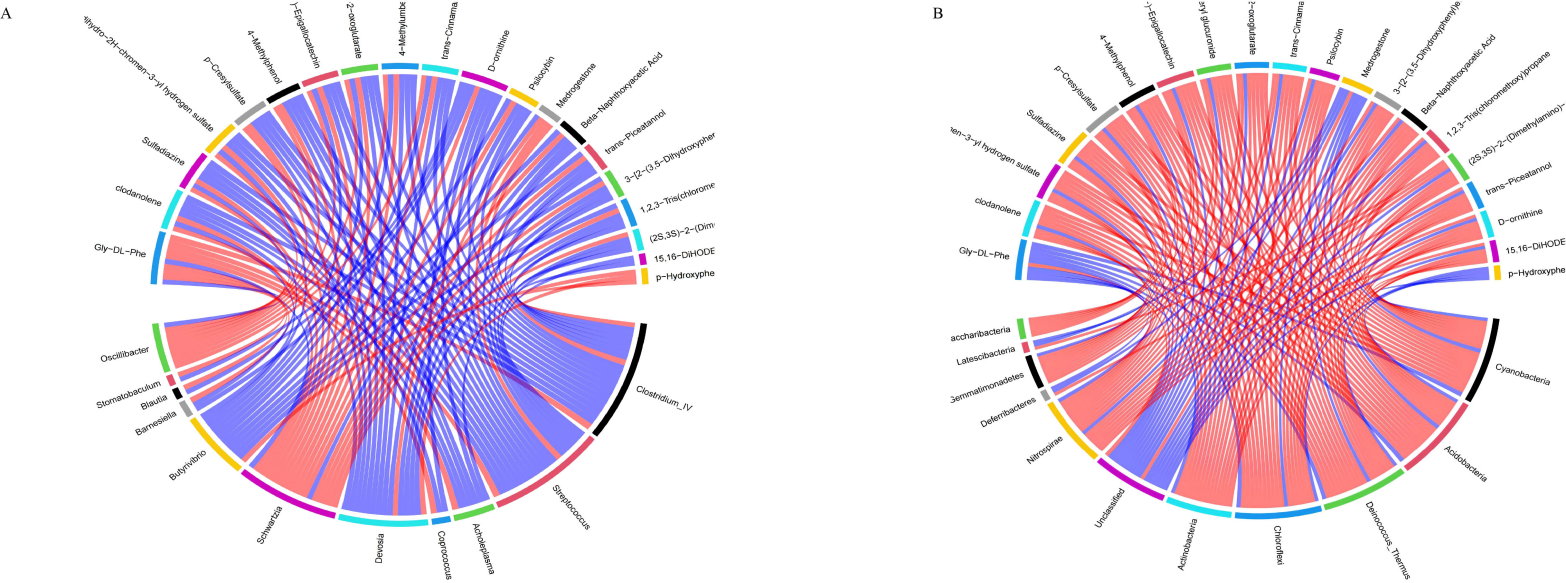
Correlation analysis between differential metabolites in serum and gastrointestinal flora. **(A)** correlation between serum differential metabolites and rumen flora, **(B)** Correlation between serum differential metabolites and intestinal flora; red and blue lines represent positive and negative correlations, respectively.

The correlation analysis results between differential metabolites in the calf serum and the level of rumen flora (**Figure 11A**). Twenty differential metabolites were strongly correlated with rumen flora (R>0.6, *P*<0.05). Metabolites such as (-)-Epigallocatechin, Trans-cinnamaldehyde, and Trans-picerol were negatively correlated with *Butyrivibrio.* Additionally, P-hydroxyphenylacetic acid was positively correlated with *Butyrivibrio*. The correlation analysis results between serum differential metabolites and intestinal flora species levels in calves (**Figure 11B**). Twenty differential metabolites were strongly correlated with the intestinal flora (R>0.6, *P*<0.05). Metabolites such as (-)-Epigallocatechin, Trans-cinnamaldehyde, and Trans-piceatannol were negatively correlated with *Oscillibacter_valericigenes* in the intestinal flora. In contrast, Glycine-DL-phenylalanine was positively correlated with *Oscillibacter_valericigenes.* These results indicate that SQBQP can regulate the abundance of gastrointestinal microflora in calves and indirectly affect the expression of serum metabolites.

## 4 Discussion

In the cattle production, some factors, including transportation, extreme temperatures, driving, and vaccination stimuli, can induce the stress response in calves, which in turn reduces their disease resistance of calves. Therefore it is of great importance to screen TCM or compound for calf healthcare according to the requirements of the ‘antibiotic reduction action’ in animal husbandry production proposed by China, to ensure the health of calves and improve breeding efficiency. The advantages of TCM include the green, natural, and non-resistant properties. This study analyzed the effects of SQBQP on ADG, blood indexes, gastrointestinal microflora, and serum metabolites of 3-month-old calves based on clinical practice. LC-MS/MS was employed to identify and quantify the active components present in SQBQP. The results showed that the primary substances in SQBQP mainly includes polysaccharides, flavonoids, and organic acids. It was demonstrated that astragalus polysaccharide (Jia et al., 2019), codonopsis pilosula polysaccharide (Gao et al., 2020), atractylodes macrocephala polysaccharide (Li et al., 2022), and saposhnikovia divaricata polysaccharide (Fan et al., 2023) could enhance the antioxidant and antibacterial capabilities. Flavonoids have been shown to confer various health benefits, exhibiting antibacterial, anti-inflammatory, anti-tumor, and antioxidant effects (Tagousop et al., 2018; Calis et al., 2020; Zeng et al., 2022). Organic acids have the effects of anti-tumor, anti-inflammatory, and free radical scavenging (Wei et al., 2013; Erukainure et al., 2017). Although the components of TCM are complex and the mechanisms of action are diverse, the evidence of their curative effect is irrefutable. TCMs such as *Radix Astragali* and *Rhizoma Atractylodis macrocephalae*, are rich in polysaccharide active ingredients (Zeng et al., 2019; Chen et al., 2020). These ingredients significantly enhance the antioxidant capacity of calves and promote their growth and development, particularly in improving the average daily weight gain of calves.

The redox state is a significant indicator of the overall health status of the body. Animals, including livestock and poultry, can rely on a variety of antioxidant enzymes to regulate the redox balance. This study found that after the administration of SQBQP on calves, there was a significant increase in the serum GSH content, a increase tendency of CAT and T-SOD content, and a decrease tendency of MDA content. These findings indicate that SQBQP can enhance the antioxidant capacity of calves. The active ingredients in TCM, such as polysaccharides and flavonoids, can activate the antioxidant system, increase the activity of antioxidant enzymes, alleviate oxidative stress and enhance the body’s antioxidant capacity by increasing serum levels of CAT, GSH, T-SOD and T-AOC and decreasing levels of MDA. (Chen et al., 2019b; Zeng et al., 2019; Wang et al., 2022b). Some studies have shown that the flavonoid eupatilin has a range of pharmacological activities, including anticancer, anti-inflammatory, antioxidant, neuroprotective, anti-allergic, and cardioprotective properties (Lu et al., 2023, 2024). Other studies have shown that ferulic acid, a naturally occurring compound with antioxidant and antimicrobial properties, improves calf growth performance by enhancing the calf’s antioxidant capacity (Peña-Torres et al., 2021). The experiment results demonstrated that SQBQP enhanced the antioxidant capacity of calves and effectively alleviated the oxidative stress.

Digestive enzymes are produced by various tissues and organs of animals and transported to target organs to decompose and digest specific substances. Cellulose is produced in the rumen of calves to break down cellulose and provide energy. Proteases, amylases, and lipases are present in the gastrointestinal tract, where they can decompose and digest nutrients, provide energy for daily requirements. The results of this study indicate that the feeding calves with SQBQP can significantly increase the activity of cellulase and amylase, while the activity of lipase is significantly reduced. Some previous research has shown that adding sunflower shells to the diet can significantly increase the amylase activity in bovine serum (Kondrashova et al., 2021). In this study, SQBQP significantly increased serum amylase activity in calves but decreased lipase activity. This suggests that serum determination can be used instead of material determination in target organs, warranting further research. Adding Bacillus subtilis to piglet diets has been shown to significantly increase the activity of amylase and lipase in the ileum (Deng et al., 2020). Substituting corn with wheat in the diet of beef cattle significantly enhances the activity of amylase, lipase, protease, and cellulase in the gastrointestinal tract (Liu et al., 2016). Naringenin affects lipid metabolism in serum primarily by directly inhibiting pancreatic lipase activity (Liu et al., 2022). In contrast, ferulic acid may indirectly affect serum digestive enzyme function by improving intestinal health and enhancing antioxidant capacity (Gong et al., 2019). The findings demonstrate that SQBQP can enhance the digestive and absorptive capabilities of calves, thereby promoting their growth by influencing serum levels of digestive enzymes.

The complex microbial flora in the gastrointestinal tract of ruminants plays a crucial role in maintaining homeostasis, regulating energy metabolism, and activating intestinal immunity (Dill-McFarland et al., 2019). By regulating feed composition, it can enhance feed utilization and animal growth performance (Jiang et al., 2021; Nguse et al., 2022; Chen et al., 2019a). This study demonstrated that SQBQP significantly impacted the regulation of rumen and intestinal microbial communities in calves and notably increased their ADG. The dominant phyla in all test samples were *Bacteroidetes, Firmicutes*, and *Proteobacteria*. In the SQBQP-H group, the abundance of *Firmicutes* in rumen fluid and feces decreased, while the relative abundance of *Bacteroidetes* in the rumen increased. Furthermore, the overall relative abundance of *Firmicutes* and *Bacteroidetes* was higher than that in the control group (C group). Both *Firmicutes* and *Bacteroidetes* are key players in the fermentation of fiber and the degradation of carbohydrates, producing various cellulases to hydrolyze macromolecular compounds such as cellulose and sugar (Jami et al., 2014; Xie et al., 2023). This process promotes fat deposition, milk fat production, and the digestion and absorption of nutrients (de Melo et al., 2023; Song et al., 2023). SQBQP has been shown to maintain the homeostasis of the rumen environment in calves by regulating the relative abundance of *Bacteroidetes* and *Firmicutes*. The increased relative abundance of *Proteobacteria* in the intestinal microflora of the SQBQP-H group was linked to the degradation and fermentation of biopolymers. Additionally, the study revealed that *Actinobacteria, Candidatus_Saccharibacteria, Deinococcus_Thermus, and Cyanobacteria* were markedly elevated in the intestinal tract of the SQBQP-H group, with *Actinobacteria* playing a pivotal role in the biodegradation of lignocellulose (Briggs et al., 2021). The study also found that the relative abundance of *Prevotella* in the rumen of the SQBQP-H group was higher than that in the C group, whereas its abundance in the intestine was lower. Flavonoids have been shown to enhance the decomposition and utilization of plant polysaccharides by increasing the relative abundance of *Prevotell* (Ghimire et al., 2022). This suggests that the increased abundance of *Prevotella* in the rumen of calves in the SQBQP-H group facilitates feed digestion and utilization. Conversely, the relative abundance of *Succinivibrio* in the rumen bacteria of the SQBQP-H group was significantly reduced, while its abundance in the intestinal bacteria was considerably increased. This indicates a competitive relationship between *Prevotella* and *Succinivibrio* in the rumen, where an increase in *Prevotella* inhibits *Succinivibrio* growth, a dynamic reversed in the intestine. *Succinivibrio* could ferment a diverse range of sugars, primarily producing acetic acid and succinic acid, with minor amounts of formic acid and lactic acid (Liu et al., 2023c). Correlation analysis results suggest a positive relationship between the abundance of *Prevotella* in rumen flora and *Succinivibrio* in intestinal flora, indicating similar roles in the different organ environments. Furthermore, correlation analysis results between gastrointestinal flora, ADG of calves, and serum indices revealed that SQBQP intervention reduced the relative abundance of *Succinivibrio* in the rumen and significantly increased the ADG of calves. After SQBQP intervention, the relative abundance of *Oscillibacte*r in the intestine was reduced, while the serum levels of CAT and T-AOC significantly increased, and the lipase levels decreased. These findings demonstrate that SQBQP exerts a positive regulatory effect on the rumen and intestinal flora of calves. An increase in the relative abundance of beneficial flora resulted in an enhanced feed digestion and absorption, subsequently improving ADG, antioxidant capacity, and the digestive efficiency in calves.

Alterations in the serum metabolite concentrations have a direct impact on the health status of animals (Guasch-Ferré et al., 2016; Wang et al., 2020; Wu et al., 2022). In this study, three down-regulated and 17 up-regulated differential serum metabolites were identified, exhibiting strong correlations with ADG and serum indicators. Among these metabolites, (-)-Epigallocatechin, Trans-cinnamaldehyde, and Trans-piceatannol were positively correlated with T-AOC, AMS, cellulase, GSH, ADG, and CAT. Conversely, hydroxyphenylacetic acid and Glycine-DL-phenylalanine were negatively correlated with these indicators. These findings suggest that alterations in the concentration of these metabolites may significantly enhance the ADG and serum indicators of calves. Numerous prospective studies have investigated the correlation between gastrointestinal microbiota and serum metabolites in both humans and animals (Zhang et al., 2021; Wang et al., 2022a). Our correlation analysis of rumen flora and serum metabolites revealed that (-)-Epigallocatechin, Trans-cinnamaldehyde, and Trans-piceatannol were negatively correlated with *Butyrivibrio*. Additionally, P-hydroxyphenylacetic acid was positively correlated with *Butyrivibrio*. Similarly, the analysis between intestinal flora and serum metabolism indicated that metabolites such as (-)-Epigallocatechin, Trans-cinnamaldehyde, and Trans-piceatannol were negatively correlated with *Oscillibacter valericigenes* in the intestinal flora, while Glycine-DL-phenylalanine was positively correlated with *Oscillibacter valericigenes*. This study found that differential metabolites showed a correlation with their corresponding gastrointestinal microbial flora in terms of significant up-regulation or down-regulation. Numerous studies have demonstrated that these metabolites can enhance animal growth performance and promote gastrointestinal health. For instance, β-naphthyloxyacetic acid is an auxin metabolite that stimulates mammalian guanylate cyclase activity. (-)-Epigallocatechin (Umme et al., 2021), Trans-cinnamaldehyde (Wang et al., 2022c), and Trans-pistol have been shown to possess anti-inflammatory and antioxidant properties, while Trans-cinnamaldehyde also exhibits antibacterial activity (Wu et al., 2020). D-ornithine, a key metabolite for energy conversion, has been observed to increase the body weight of young goats (Wang et al., 2023). SQBQP, a traditional Chinese medicine compound, has demonstrated antioxidant, anti-inflammatory, and antibacterial properties. This study found that SQBQP intervention in calves significantly up-regulate the content of these metabolites in calf serum, reduced the relative abundance of *Butyrivibrio* in rumen bacteria, and *Oscillibacter_valericigenes* in intestinal bacteria. Further studies revealed that SQBQP can up-regulate calf serum metabolites, affect various metabolic pathways, including amino acid biosynthesis, β-alanine metabolism, phenylalanine, tyrosine and tryptophan biosynthesis, protein digestion and absorption, and ABC transporter pathways. Amino acid biosynthesis plays a pivotal role in protein synthesis, energy provision, and participation in the metabolic pathways. Through conjugated and independent pathways, organisms can convert inorganic substances into amino acids, regulate by feedback inhibition and gene regulation. Prenatal nutrient supplementation significantly affected β-alanine metabolism in pregnant cows (Schalch Junior et al., 2022). Moreover, yam has been found to regulate abnormal fecal flora caused by diarrhea in rats and participate in β-alanine metabolism, as well as phenylalanine, tyrosine, and tryptophan biosynthesis (Zhang et al., 2023). Protein digestion and absorption, along with ABC transporters are the crucial metabolic pathways for calf gastrointestinal health (Huang et al., 2020). Tyrosine metabolism is also vital for the normal metabolism of perinatal dairy cows (Lopreiato et al., 2023; Zhao et al., 2024). Combining weight gain, serum partial indicators, microbiome, metabolomics, and their correlation analysis, it was confirmed that SQBQP promotes gastrointestinal health and increases the average daily gain of calves by regulating the abundance of gastrointestinal flora, serum metabolites, and metabolic pathways.

## 5 Conclusion

The study demonstrated that SQBQP could enhance the ADG of calves, improve serum indices, regulate the relative abundance of gastrointestinal flora, and influence the serum metabolite changes in calves. Further correlation analysis of the gastrointestinal microbiome and metabolomics revealed that SQBQP could enhance the body weight and antioxidant capacity of calves by regulating the relative abundance of gastrointestinal microbial flora, including *Succinivibrio*, *Butyrivibrio*, and *Oscillibacter_valericigenes*, and the levels of metabolites such as Trans-cinnamaldehyde, (-)-Epigallocatechin, Trans-piceatannol, P-hydroxyphenylacetic acid, and Glycine-DL-phenylalanine. Moreover, the study identified specific bacterial species, namely *Butyrivibrio* and *Oscillibacter_valericigenes*, and key metabolites including Trans-cinnamaldehyde, (-)-Epigallocatechin, Trans-piceatannol, P-hydroxyphenylacetic acid, and Glycine-DL-phenylalanine, which were found to promote gastrointestinal health in calves. These results provided the preliminary evidence of the potential of SQBQP as a functional feed additive. However, further systematic research and long-term monitoring are needed to fully evaluate the potential for the widespread use.

## Data Availability

The datasets used and analyzed during the current study are available from the corresponding author upon reasonable request.
